# One maternal lineage leads the expansion of *Thaumastocoris peregrinus* (Hemiptera: Thaumastocoridae) in the New and Old Worlds

**DOI:** 10.1038/s41598-020-60236-7

**Published:** 2020-02-26

**Authors:** Dayanna do N. Machado, Ervandil C. Costa, Jerson V. C. Guedes, Leonardo R. Barbosa, Gonzalo Martínez, Sandra I. Mayorga, Sergio O. Ramos, Manuela Branco, André Garcia, Juan Manuel Vanegas-Rico, Eduardo Jiménez-Quiroz, Stefania Laudonia, Tania Novoselsky, Donald R. Hodel, Gevork Arakelian, Horacio Silva, Clérison R. Perini, Ivair Valmorbida, Gustavo A. Ugalde, Jonas A. Arnemann

**Affiliations:** 1 Doutoranda pelo Programa de Pós-Graduação em Engenharia Florestal, Universidade Federal de Santa Maria, Santa Maria, Brasil; 2 Departamento de Defesa Fitossanitária, Avenida Roraima n. 1000, prédio 42, sala 3223, 97105-900 Santa Maria, Rio Grande do Sul Brasil; 3 Empresa Brasileira de Pesquisa Agropecuária – Embrapa Florestas, Colombo, Paraná, 83411-000 Brazil; 4 Instituto Nacional de Investigación Agropecuaria (INIA), Ruta 5 Km 386, Tacuarembó, Uruguay; 5 Servicio Agrícola y Ganadero (SAG), Av. Presidente Bulnes 140, Santiago, Chile; 6 Instituto Nacional de Tecnología Agropecuaria (INTA), Estación Yuquerí, Ruta Provincial 22 y vías del Ferrocarril 3200, Concordia, Entre Ríos Argentina; 7 Centro de Estudos Florestais, Instituto Superior de Agronomia, Universidade de Lisboa, Lisboa, Portugal; 8 Laboratorio de Control de Plagas, Unidad de Morfología y Función (UMF), Facultad de Estudios Superiores Iztacala, UNAM. Av. de los barrios #1. Los Reyes Iztacala, Tlalnepantla de Baz, 54090 Mexico; 9 Laboratorio de Análisis y Referencia en Sanidad Forestal, Av. Progreso 3, 04100 Coyoacán Ciudad de México, Mexico; 10 Dipartimento di Agraria, Università degli Studi di Napoli Federico II, Portici, Italy; 11 The Steinhardt Museum of Natural History, Israel National Center for Biodiversity Studies, Tel Aviv University, Tel Aviv, 69978 Israel; 12 University of California, Cooperative Extension, 700 W. Main Street, Alhambra, California 91801 United States of America; 13 Entomologist, Los Angeles County Agricultural Commissioner, 11012 S. Garfield Ave, South Gate, CA 90280 United States of America; 14 Faculdad de Agronomía Universidad de la República Uruguay, Ruta 3 km 363, 60000 Paysandú, Uruguay; 15 Department of Entomology, Iowa State University, Ames, Iowa USA

**Keywords:** Biological techniques, Molecular biology

## Abstract

The bronze bug, *Thaumastocoris peregrinus*, an Australian native insect, has become a nearly worldwide invasive pest in the last 16 years and has been causing significant damage to eucalypts (Myrtaceae), including *Eucalyptus* spp. and *Corymbia* spp. Its rapid expansion leads to new questions about pathways and routes that *T. peregrinus* used to invade other continents and countries. We used mtDNA to characterize specimens of *T. peregrinus* collected from 10 countries where this species has become established, including six recently invaded countries: Chile, Israel, Mexico, Paraguay, Portugal, and the United States of America. We then combined our mtDNA data with previous data available from South Africa, Australia, and Europe to construct a world mtDNA network of haplotypes. Haplotype A was the most common present in all specimens of sites sampled in the New World, Europe, and Israel, however from Australia second more frequently. Haplotype D was the most common one from native populations in Australia. Haplotype A differs from the two major haplotypes found in South Africa (D and G), confirming that at least two independent invasions occurred, one from Australia to South Africa, and the other one from Australia to South America (A). In conclusion, Haplotype A has an invasion success over many countries in the World. Additionally, analyzing data from our work and previous reports, it is possible to suggest some invasive routes of *T. peregrinus* to predict such events and support preventive control measures.

## Introduction

The eucalypts represent over 20% of commercial tree plantations worldwide^1^ and are also common in many landscapes. However, in recent years the significant expansion of planted areas has also increased the risk of introduction of invasive pest species^2,3^. From 1870 to 2014, at least 42 eucalypt insect pests were introduced outside their native ecosystems, notably of the order Hemiptera (17 species)^1^. Among the various invasive pests of eucalypts, the bronze bug, [*Thaumastocoris peregrinus* Carpintero & Dellapé 2006 (Hemiptera: Thaumastocoridae)]^4^, has become the most widespread. In areas of recent colonization, *T. peregrinus* caused significant economic and esthetic damage to eucalypt trees. Symptoms of the insect's attack include leaf discoloration ranging from chlorosis to silver, bronze, and finally brown at defoliation, decreasing photosynthetic area and leading to the death of young trees^5–8^.

*Thaumastocoris peregrinus* was firstly detected as invasive in South Africa in the year 2003^9^ and shortly thereafter in Argentina in 2005^4,10^. A few years later, it was found in other African and South American countries, and later also in North America, Europe, and Israel (Table 1). The EPPO (European Plant Protection Organization) added *T. peregrinus* to the EPPO alert list in 2012, due to its invasive behavior and its potential damage to eucalypt trees^11^.

**Table 1:** First records of *Thaumastocoris peregrinus* outside Australia.

Country	Year	References
**Africa**		
South Africa	2003	^9^
Zimbabwe	2007	^82–84^
Malawi	2008	^82^
Kenya	2009	^82,83^
Reunion Island	2013	^23^
Uganda	—	^82^
Tanzania	—	^82^
Rwanda	—	^82^
Mozambique	—	^42^
**South America**		
Argentina	2005	^4,10^
Brazil	2008	^7^
Uruguay	2008	^85^
Chile	2009	^63^
Paraguay	2010	^86^
**Oceania**		
New Zealand	2012	^87^
**Mediterranean Basin**		
Italy	2011	^66,88,89^
Portugal	2012	^54^
Israel	2014	^90^
Spain	2014	^70^
Albania	2016	^71^
Greece	2017	^72^
**North America**		
Mexico	2015	^76^
United States of America	2016	^77,78^

Increasing economic globalization has facilitated the dispersal of *T. peregrinus* and other invasive pests^1^. The intensification in intercontinental goods and passenger transport has significantly contributed to the introduction of invasive insects^12–14^. DNA barcoding can support studies of invasive pests and its possible routes of invasion as well as provide accurate species identification. Furthermore, analysis of the variability within a standard DNA barcode region can also aid determining potential origin and pattern of dispersal of an invasive pest^15–18^. Previous analysis of mtDNA (partial COI region) from South African and South American populations of *T. peregrinus* revealed the presence of three haplotypes and more than one route of dispersal from Australia to Africa and South America were proposed^16^. The molecular characterization of *T. peregrinus* populations in the newly invaded areas was lacking. Thus, we sequenced part of the mitochondrial gene of Cytochrome C Oxidase subunit I (COI) of *T. peregrinus* collected in different countries. Possible invasion routes for each country, based on data from the literature and collected in the present work, are analyzed and discussed.

## Material and Methods

### Sampling procedure

**Figure 1:**
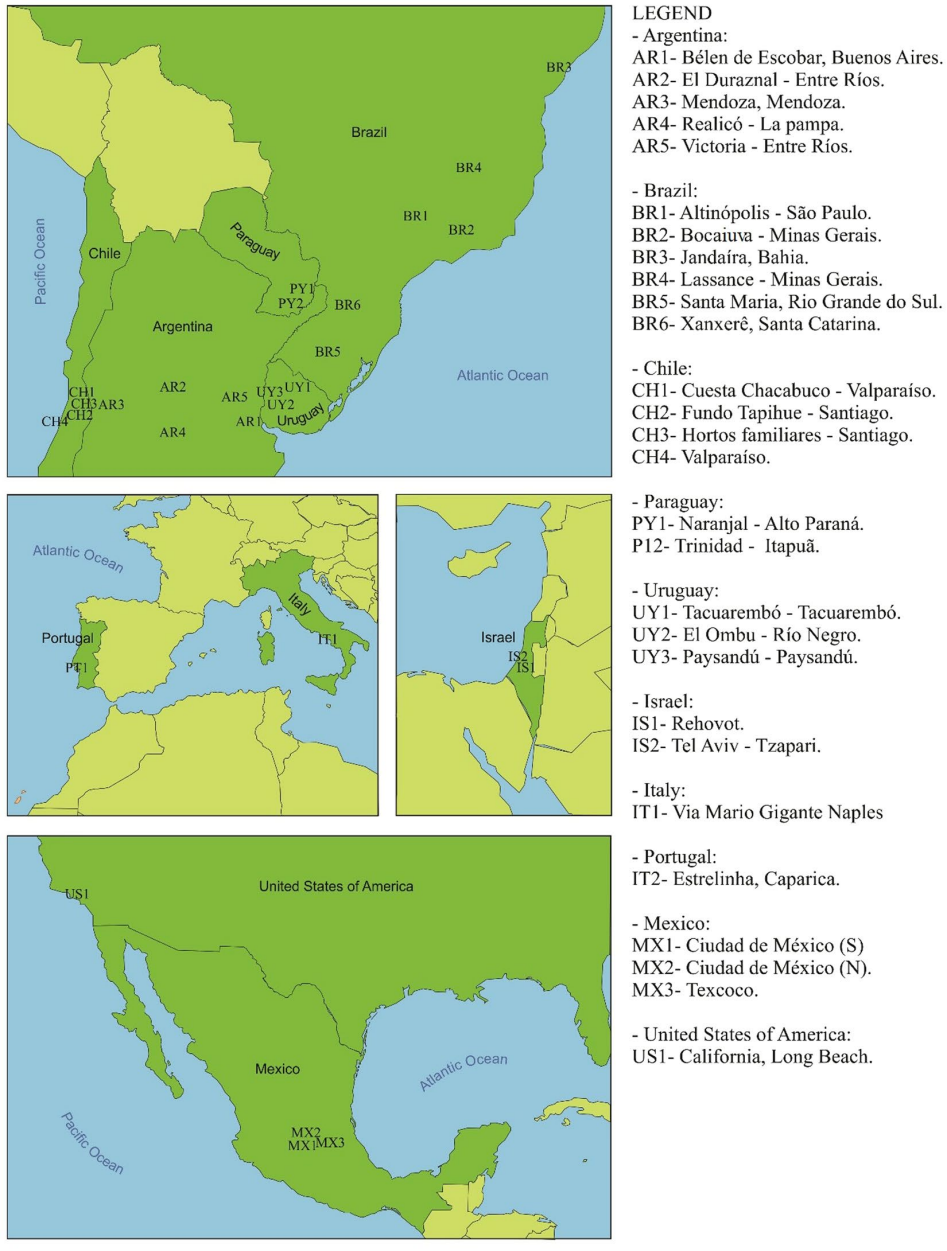
Locations where the specimens of *Thaumastocoris peregrinus* were collected in South America, North America, Europe, and Israel.

Specimens of *T. peregrinus* were randomly collected from *Eucalyptus* trees in five countries in South America (Argentina, Brazil, Chile, Paraguay, and Uruguay), two in North America (the USA and Mexico), one in the Middle East (Israel) and two in Europe (Italy and Portugal) (Fig. 1). At each site, adults of *T. peregrinus* were manually detached from branches and/or leaves using a paintbrush or aspirator and placed into 1.5 mL vials containing 98% ethanol to preserve their DNA. Approximately 300 specimens of *T. peregrinus* were collected at 28 sampling sites from November 2016 to May 2018. Each vial was labeled with the municipality, date, and coordinates of the collection site. Vials from all sample collection sites were sent to the Biotechnology Laboratory of the Federal University of Santa Maria, Santa Maria, RS, Brazil, where they were stored at −20 °C until genomic DNA extraction.

### DNA extraction, PCR amplification, and COI-gene sequencing

The identification of all *T. peregrinus* specimens was confirmed based on morphological characters^4^. DNA extraction was performed individually for each specimen using the DNeasy Blood and Tissue Kit (Qiagen, Hilden, Germany) according to the manufacturer's instructions. Depending on the availability of insects and the proximity of sampling sites, we used 1 to 10 insects per location for DNA extraction (Table 2). Each adult insect was removed from the vial with ethanol and left to air dry on a paper towel. The entire body was individually macerated in a 1.5 mL tube containing 180 µL of buffer ATL and 20 µL of proteinase K and incubated at 56 °C for 12 hours. Subsequently, genomic DNA was purified in a silica-based matrix and eluted in 35 µL of buffer AE. The concentration of DNA was assessed in a spectrophotometer (NanoDrop™ 1000, Thermo Scientific, Wilmington, DE, USA).

**Table 2:** Number of individuals used/sequenced by population.

Country	Populations	Number of individuals sequenced	Date of collection	Latitude	Longitude	Haplotypes	Reference
Australia	Sydney	118	06/22/2005	34°01′39″S	151°04′01″E	V, M, T, E, C, H, G, D, A	^20^
	Chelmsford	17	05/11/2008	26°15.014′S	151°49.359′E	O, TB, F, B, CB, D	^20^
	Coonabarabran	04	04/10/2004	31°16′S	149°17′E	M, A, D	^20^
	Dubbo	09	03/17/2004	32°14′38″S	148°36′32″E	VB, QB, OB, SC, B, D	^20^
	Nyngan	05	07/08/2010	31°33′35″S	147°11′39″E	W, P, D	^20^
	Cootamundra	05	03/23/2006	34°38′S	148°02′	V, Q, CE, A, D	^20^
	Narrandera	02	04/20/2006	34°45′S	146°33′E	X, D	^20^
	Canberra	11	07/26/2010	35°16′31″S	149°07′28″E	ZC, ZB, NB, XB, D	^20^
	Wagga Wagga	01	03/22/2006	35°07′S	147°22′E	P	^20^
	Seymour	03	07/18/2010	37°01′15″S	145°07′29″E	XC, SC, D	^20^
	Bacchus Marsh	03	07/16/2010	37°40′28″S	144°26′15″E	IB, BC	^20^
	Ararat	04	07/16/2010	37°16′59″S	142°55′41″E	TC, R, JB, D	^20^
	Renmark	06	07/10/2010	34°10′30″S	140°45′05″E	Z, SC, SB, LB, K, HB	^20^
	Olary	08	07/09/2010	32°16′53″S	140°19′36″E	S, UB, UC, U, Y	^20^
	Mitcham	01	07/14/2010	34°59′20″S	138°37′39″E	L	^20^
Reunion Island	KT273623	01	04/2015	—	—	D	^23^
	KT273624	01	04/2015	—	—	D	^23^
	KT273625	01	04/2015	—	—	D	^23^
	KT273626	01	04/2015	—	—	D	^23^
	KT273627	01	04/2015	—	—	D	^23^
Italy	KF437485	01	—	—	—	IT	^21^
Spain	MN401749	10	04/2019	40°25′07.8′′N	3°41′13.2′′W	A	^22^
South Africa	32 localities	62	2006–2008	—	—	D, G	^16^
South America	Two Argentina, three Brazil, single Uruguay	32	2007–2008	—	—	A	^16^
Argentina	Bélen de Escobar	05	02/22/2017	34°18′52′′S	58°52′11′′O	A	This study
	El Duraznal	03	02/09/2017	31°16′29′′S	58°14′55′′O	A	
	Mendoza	03	02/15/2017	32°52′58′′S	68°52′56′′O	A	
	Realicó	02	02/21/2017	35°03′13′′S	64°06′54′′O	A	
	Victoria	02	02/13/2017	32°37′16′′S	60°10′20′′O	A	
Brazil	Altinópolis, SP	05	11/20/2016	21°05′25′′S	47°25′37′′O	A	This study
	Bocaiuva, MG	01	01/13/2017	—	—	A	
	Jandaira,	05	02/08/2017	11°35′10′′S	37 °46′16′′O	A	
	Lassance, MG	04	11/15/2016	18°02′44′′S	44°48′38′′O	A	
	Santa Maria, RS	05	12/22/2016	29°43′08′′S	53°42′51′′O	A	
	Xanxerê, SC	05	02/15/2017	26°89′47′′S	52°46′15′′O	A	
Chile	Cuesta Chacabuco	03	02/10/2017	34°29′42′′L	63°54′26′′N	A	This study
	Fundo Tapihue	03	02/07/2017	32°06′03′′L	63°33′50′′N	A	
	Hortos familiares	02	02/07/2017	33°18′73′′L	63°31′12′′N	A	
	Hahuel	02	02/10/2017	34°87′94′′L	63°81′88′′N	A	
Paraguay	Naranjal	05	04/07/2017	25°59′30′′S	55°07′17′′O	A	This study
	Trinidad	05	04/07/2017	27°08′04′′S	55°43′47′′O	A	
Uruguay	El Ombu	02	02/10/2017	32°58′11′′S	57°41′58′′O	A	This study
	Paysandú	05	02/22/2017	32°22′47′′S	58°03′12′′O	A	
	Tacuarembó	03	02/22/2017	31°44′20′′S	55°58′47′′O	A	
Mexico	Mexico City (N)	04	08/20/2017	19°27′47′′N	99°04′19′′O	A	This study
	Mexico City (S)	03	08/20/2017	19°17′51′′N	99°05′50′′O	A	
	Texcoco	03	08/25/2017	19°29′38′′N	98°53′37′′O	A	
United States of America	Long Beach	02	07/17/2016	33°49′51′′N	118°06′56′′O	A	This study
Israel	Gan Le’ummi Yarqon	10	05/14/2018	32°05′59′′N	34°48′26′′E	A	This study
	Rehovot	04	05/14/2018	31°52′22′′N	34° 49′30′′E	A	
Italy	Via Mario Gigante	10	10/24/2017	40°50′0.8′′N	14°11′38′′L	A	This study
Portugal	Caparica	10	07/01/2017	38°38′43′′N	09°12′51”O	A	This study
Total		423					

Mitochondrial DNA sequences from this study were combined with other sequences deposited in Genbank^16,21–23^ and some not deposited in Genbank^20^.

A fragment of the mitochondrial COI gene (468 bp) was amplified from 116 individuals through polymerase chain reaction (PCR) using the primers Tp2390F (5′ACCCGAGCATACTTTACTTC) and Tp2937R (5′ATTGTGGCTCGTTTTGATA)^16^. Each PCR reaction was performed with a final volume of 12 µL composed by 1.25 µL of JumpStart^TM^ 10X reaction buffer; 0.625 µL de dNTP mix (10 nM of each); 1.0 µL of each primer (10 pM), 0.220 µL of JumpStart^TM^ DNA Polymerase (2.5 U/µL) (Sigma-Aldrich, St. Louis, MO, USA); 1 µL de template DNA (05–100 ng/µL) and 7.0 µL of ultra-pure water.

PCR amplification consisted of an initial denaturation step at 98 °C for 30 s, followed by 30 cycles at 95 °C for 30 s, 48 °C for 30 s and 72 °C for 1.5 min, and a final extension at 72 °C for 10 min^16^. Amplified products were resolved on 1.0% agarose electrophoresis gel, pre-stained with Nancy-520 DNA gel stain (Sigma-Aldrich) and visualized using a gel documentation system. Successfully amplified PCR products were sequenced by ACTGene Molecular Analyses (Alvorada, RS, BR), using the BigDye Terminator method on an ABI 3500 Genetic Analyzer (Applied Biosystems, Foster City, CA, USA).

### Data analysis

Quality assessment, trimming, editing, and analysis of each DNA sequence were performed using the software Pregap and Gap4 within the Staden package^19^. CLC Sequence Viewer (Version 7.8.1-QIAGEN Aarhus A/S) was used to retrieve and align sequences with a length of 468 bp. A pBLAST analysis (amino acid homology confirmation) with *T. peregrinus* partial mtDNA COI genes deposited on the NCBI (National Center for Biotechnology Information, USA) database was also performed.

The 116 COI sequences generated in this study were combined with 197 sequences of 16 populations from Australia^16,20^. We also used 32 sequences from South America, 62 from South Africa^16^, 1 from Italy (KF437485)^21^, 10 from Spain (MN401749)^22^, and 5 from Reunion Island (France) (GenBank − KT273623, KT273624, KT273625, KT273626, and KT273627)^23^. The inference and visualization of genetic relationships among intraspecific sequences used to generate a haplotype network were conducted using TCS network^24^ within the program PopART^25^.

## Results

Fragments of mtDNA from 116 individuals of *T. peregrinus* from 28 populations were amplified and sequenced, resulting in a trimmed sequence of 468 pb of the COI gene. Samples of *T. peregrinus* from six countries (Chile, Paraguay, Mexico, Portugal, Israel, and the USA) were sequenced for the first time. For all the sampled sites from South America (Chile, Paraguay, Argentina, Brazil, and Uruguay), North America (Mexico and the USA) and the Mediterranean basin (Italy, Portugal, and Israel), we confirmed the presence of a unique haplotype (haplotype A).

**Figure 2:**
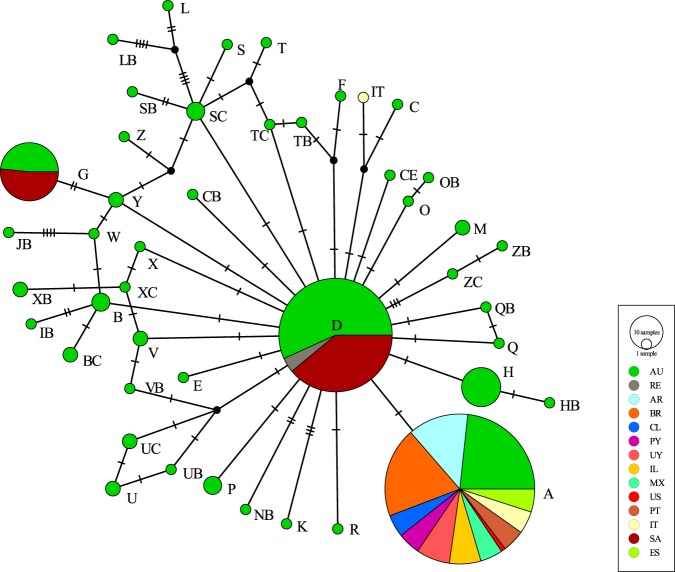
Mitochondrial haplotype network. Circle sizes are in approximate proportion to haplotype frequencies. Each mark represents one nucleotide difference. Countries: Australia (AU); Reunion Island (RE); Argentina (AR); Brazil (BR); Chile (CL); Paraguay (PY); Uruguay (UY); Israel (IL); Mexico (MX); United States of America (US); Portugal (PT); Spain (ES); Italy (IT); South Africa (SA).

Combining our data with sequences not deposited in GenBank^20^ and with others previously deposited in this database (Table 2), we obtained a total of 423 *T. peregrinus* COI sequences and 45 unique haplotypes were identified. The proportions of the different haplotypes among the countries are presented on a haplotype network (Fig. 2). This network reveals that the greatest diversity of haplotypes is from the native region of *T. peregrinus* in Australia. Haplotype D is the ancestral one and all the other haplotypes radiate from it (Fig. 2). Haplotype D was the most frequent haplotype in Australia and found in 10 sites^20^. Haplotype A was present in three of 10 sites where haplotype D was also reported: Sydney, Coonabarabran, and Cootamundra^20^. The difference between the two haplotypes is only one base pair in the partial mtDNA COI fragment (468 bp)^16^ and possibly represents a silent mutation.

**Figure 3:**
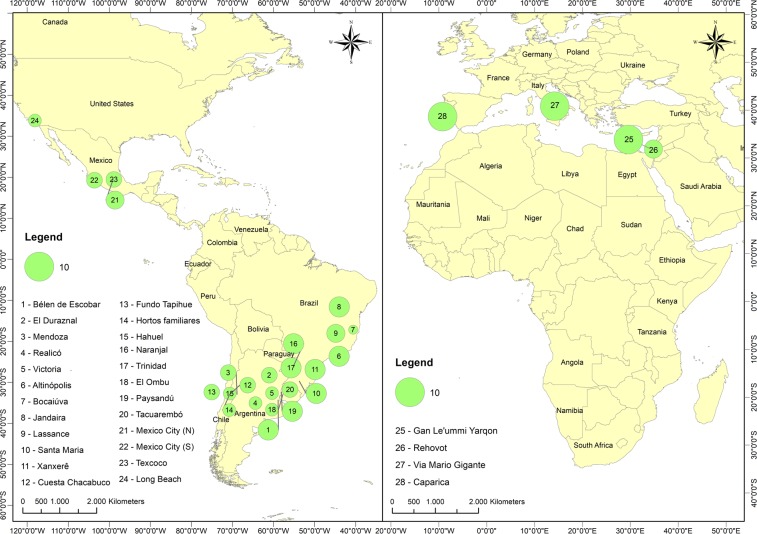
Sampling sites (28) and distribution of haplotype A in Americas, Europe and Asia. Numbers of sites related to each country: Argentina (1–5); Brazil (6–11); Chile (12–15); Paraguay (16–17); Uruguay (18–20); Mexico (21–23); United States of America (24); Israel (25–26); Italy (27) and Portugal (28).

**Figure 4:**
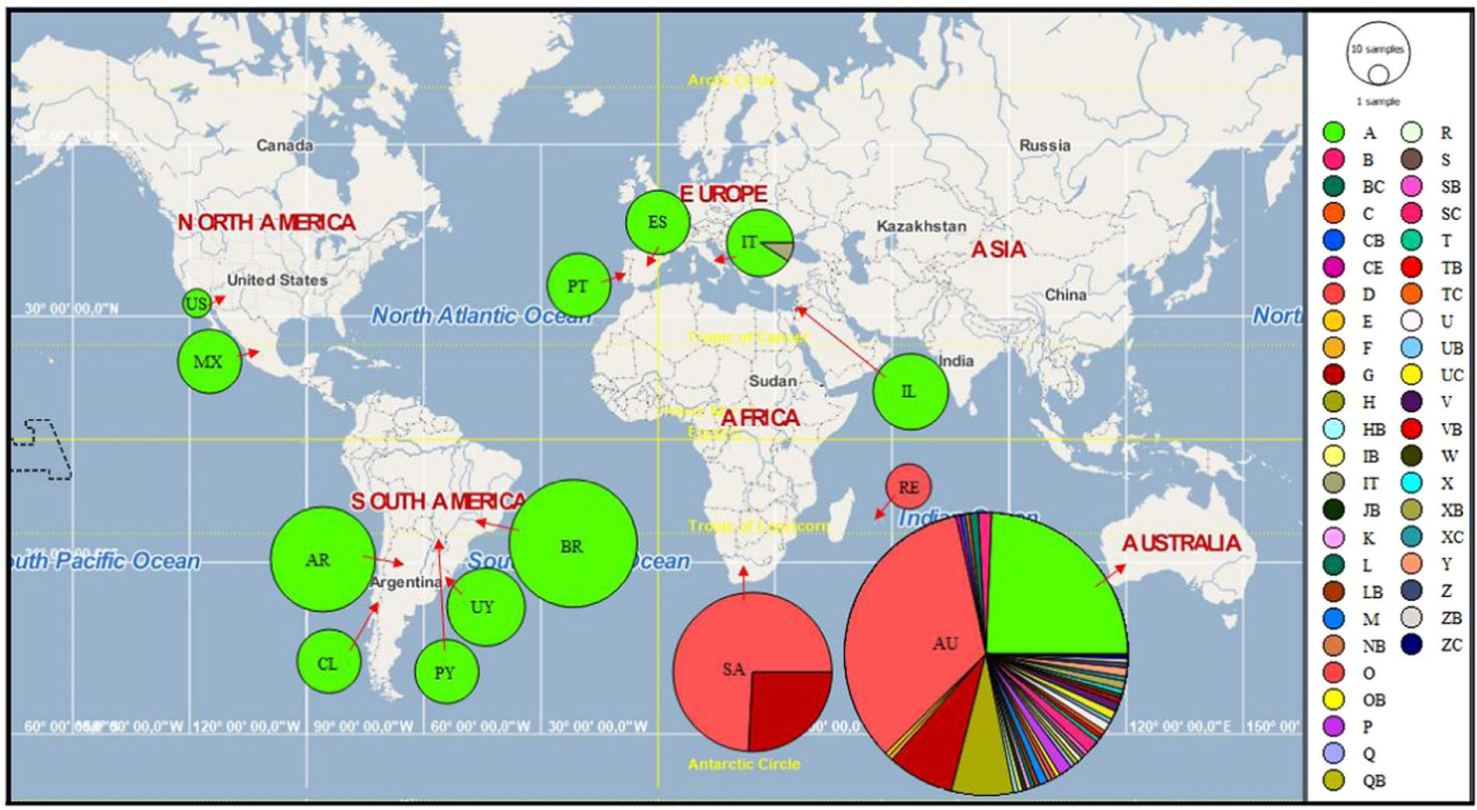
Haplotype distribution of *T. peregrinus* in 14 countries. Australia (AU); Reunion Island (RE); Argentina (AR); Brazil (BR); Chile (CL); Paraguay (PY); Uruguay (UY); Israel (IL); Mexico (MX); United States of America (US); Portugal (PT); Spain (ES); Italy (IT); South Africa (SA). The size of pie charts is relative to the number of individuals sequenced at each locality.

Outside of Australia, only four haplotypes were found: D and G in South Africa^16^, IT in Italy^21^, and A in South America^16^, North America, Europe, and Asia. From our work, haplotype A was the most frequent and widespread, found at 28 sites (116 sequences), in three populations from Australia (48 sequences)^20^, six populations in South America (32 sequences)^16^ and one population in Spain (10 sequences)^22^, comprising a total of 206 sequences (Fig. 2). Based on the combined sequences, haplotype D was found in Australia (67 sequences)^16,20^, Reunion Island (five sequences)^23^, and in nearly all the sites sampled in South Africa (46 sequences)^16^, making a total of 118 sequences. The third and fourth most frequent haplotypes were G and H, 31 sequences (16 for South Africa and 15 for Australia) of G and 14 sequences (all in Australia) of H, totaling 45 sequences^16,20^. All the remaining haplotypes were represented by three or fewer individuals. We note that all haplotypes found in the invaded regions were also found in their naturally occurring regions in Australia, except for the IT haplotype, which was found only in Italy (Genbank KF437485)^21^ (Fig. 2). To understand and to have a wide view of these results properly, the Fig. 3 shows the haplotype A distribution at our sampling sites in Americas, Europe and Israel (10 countries). In addition, the Fig. 4 represent a world haplotype distribution from 14 countries and 423 sequences.

## Discussion

### Genetic diversity

Our study updates information on the genetic diversity and mtDNA haplotype distribution of *T. peregrinus* worldwide. Samples from several locations in seven countries, including newly invaded countries in South America (Chile and Paraguay), North America (the USA and Mexico) and the Mediterranean Basin (Portugal, Italy, and Israel) were collected and analyzed. We also combined our mtDNA data with previously available sequences of *T. peregrinus*^16,20–23^ (Table 1). Our results demonstrate that haplotype A is predominant in South America, North America, Europe, and Israel, indicating a low genetic diversity of *T. peregrinus* outside its native region.

In contrast with the low genetic diversity found in the invaded areas, the Australian populations of *T. peregrinus* (the native range of the species) revealed a high mtDNA COI haplotype diversity^16,20^. Genetic diversity is expected to be higher in a species native range in comparison with invasive populations^26^, possibly because of bottleneck effects during the invasion process. The newly introduced species might be reduced to a few individuals because of environmental events (e.g. hurricane, forest fires) and anthropic actions, resulting in low genetic diversity^27^. Furthermore, genetic drift and the founder effect might also account for the low genetic diversity of the new invasions^28^. Selection pressure on a given population of the invasive species, that allow the survival of the fittest insects, might result in low genetic diversity during the first decades of species establishment^29^. These evolutionary forces associated with the adaptability of haplotype A to different environments seem to have driven the expansion of *T. peregrinus* worldwide, compared to the haplotypes D and G.

Low genetic diversity has also been reported for many other forest invasive pests. For example, populations of *Anoplophora glabripennis* (Motschulsky) (Coleoptera: Cerambycidae)^30^, *Leptocybe invasa* Fisher & La Salle, 2004 (Hymenoptera: Eulophidae)^31,32^, *Solenopsis invicta* Buren, 1972 (Hymenoptera: Formicidae)^33^, *Solenopsis geminata* (Fabricius, 1804) (Hymenoptera: Formicidae)^34^, and *Sirex noctilio* Fabricius, 1793 (Hymenoptera: Siricidae)^35^ demonstrated low genetic diversity in areas where these species are considered invasive. In a new environment, invasive populations can take several years to increase their genetic diversity^36,37^.

### Possible invasion routes of *T. peregrinus* in South America, Europe, North America, and Asia

Knowledge of possible ways by which an insect can invade a new area and about its biology is essential to identify routes of invasion and to develop and adopt effective strategies to prevent and/or manage additional introductions^1,38^. Possible routes of invasion of eucalypts pests include active dispersion, passive transport by biotic and abiotic agents, or accidental transport^39^. Even though our data are limited to a partial mtDNA COI fragment (468 bp) from *T. peregrinus*, an invasive range can be projected using mtDNA which possesses a unique haplotype signature of a population as stated by Goldstein *et al*.^40^. The mtDNA is inherited most often only from the mother and does not undergo recombination^41^, enabling to assess the number of genetically unique founder females^42–45^. Studies of invasive species used similar methodologies, for example of *T. peregrinus*^16^, wood-boring beetles^46^, *Melanagromyza sojae* (Zehntner, 1900) (Diptera: Agromyzidae)^47^ and *Helicoverpa armigera* (Hübner, 1808) (Lepidoptera: Noctuidae)^48^.

**Figure 5:**
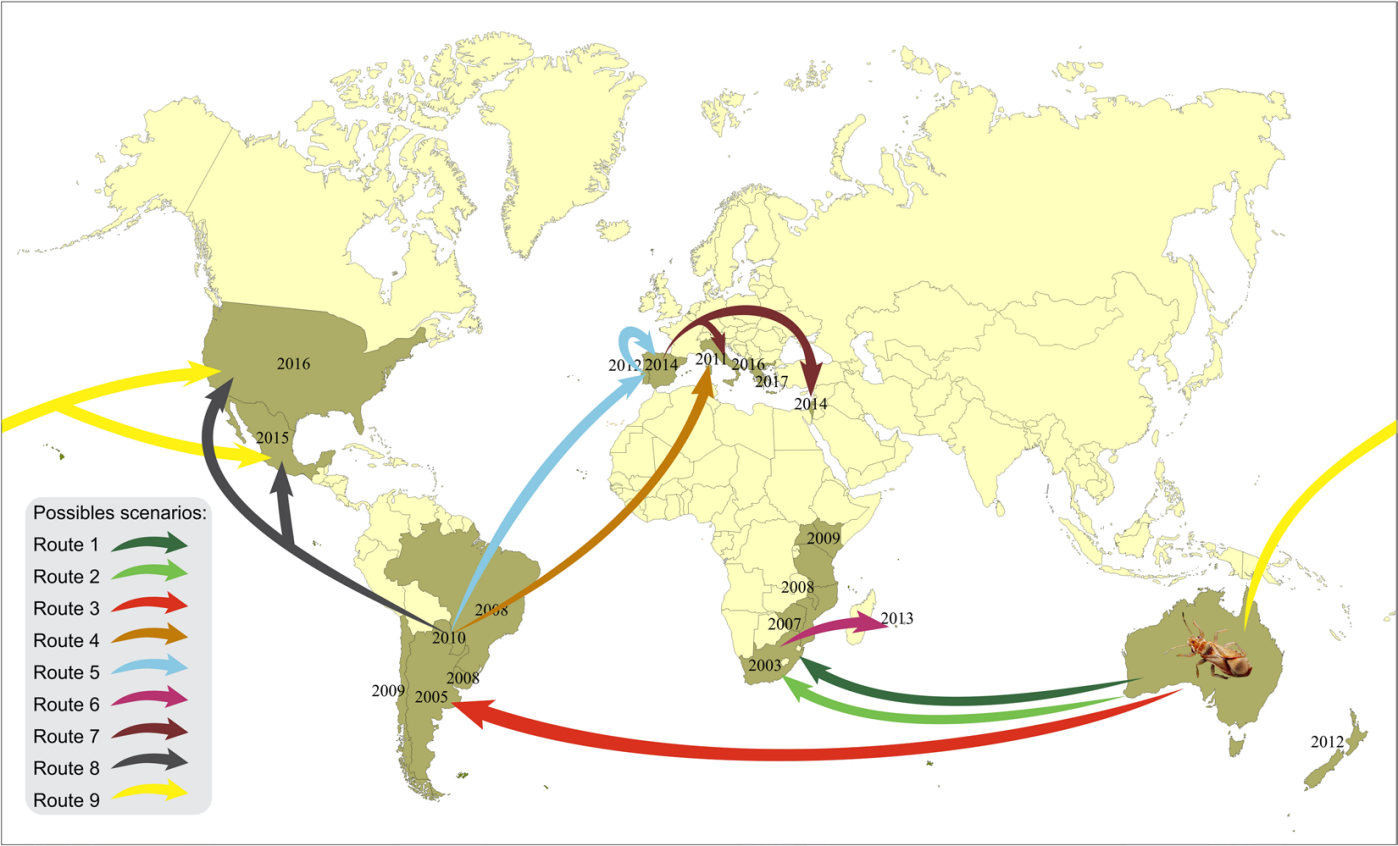
Possible invasion and dispersal routes of *T. peregrinus* in the New and Old World.

Using data collected in our work and from the literature, we suggest nine possible invasion routes of *T. peregrinus* (Fig. 5). These conclusions are based on (1) spatio-temporal events; (2) previous report of invasion routes of each country; (3) movement of people, goods, and timber through motorways and waterways between countries; and (4) our findings that analyzed 116 sequences from specimens collected in countries in South and North Americas, Europe, and Asia.

Routes 1 and 2: In 2003, *T. peregrinus*^4^ was firstly reported in South Africa, erroneously identified as *Thaumastocoris australicus* Kirkaldy^9^. Years later, a study using DNA barcoding in specimens of *T. peregrinus* from South Africa revealed the presence of two haplotypes, D (route 1) and G (route 2), indicating that the introduction happened in two distinct invasion events, both aided by transport of people and goods primarily through airline with flights from Australia (Sydney)^16^. Further studies should update the *T. peregrinus* haplotypes present in Africa because its occurrence has increased throughout the continent, possibly as a consequence of new invasions or further dispersal of populations already present in the invaded area.

Route 3: *T. peregrinus* was first reported in the Americas in Argentina in 2005^4^. This invasion was an independent event of haplotype A directly from Australia and possibly from Sydney since the haplotype was different from that of the South African invasion^16^. Geographical proximity, land border, surface transport of people and goods, and the presence of extensive eucalypt plantations alongside highways margins in Argentina, Brazil, Paraguay, and Uruguay facilitated the spread of this invasive pest through South America. The monitoring data of *T. peregrinus* in Brazil suggest its entrance via airport^8^. In Chile, the invasion of *T. peregrinus* was thought to be passive and was spread by different means, including trucks, buses and ships, and other vectors, mainly birds^49,50^. Natural dispersion by flight is also possible due to Argentina's proximity to other countries.

The geographical position of a country can influence and help to predict the number of invasive insects, which is related to the length and nature of borders with neighboring countries and correlations among land area, population, and gross domestic product^1,51^. However, some factors may be more likely than others, as is the case of eucalypt pest introductions into New Zealand from Australia, probably due to the proximity and frequent trade between the two countries^1,52^.

Route 4: In Europe, the first reported occurrence of *T. peregrinus* was in Italy in 2011, possibly introduced through the transport of timber from South America and South Africa^53^. However, this hypothesis was not based on the population genetic signatures. In 2014, mtDNA molecular characterization of *T. peregrinus* specimens from Italy was performed, and a haplotype called IT was identified^21^. Strangely, haplotype IT has not been reported from Australia and several factors might offer an explanation, including a not yet detected haplotype with unusual low frequency in Australia, or a new haplotype restricted to Italy or from an undetermined population along the invasive routes between Australia and Italy. Additionally, our results confirm the presence of haplotype A in Italy, suggesting an invasive pathway from South America because of wood transport between the two countries. This scenario is more likely than an introduction from South Africa as previously reported^53^. Nonetheless, based on the presence of haplotype A in Portugal and recently in Spain^22^, we also hypothesize dispersion and incursions from many of these countries into Italy because of the short geographical distance, the land border, and the motorway connections between countries (Portugal, Spain, France, and Italy), and intense movement of tourists and goods.

Route 5: The second country in Europe to report an invasion of *T. peregrinus* was Portugal in 2012^54^. Based on South America's status as the main supplier of eucalypts wood to Europe^55,56^, South America has been suggested as a route of invasion of *T. peregrinus* into Europe^54^. Also, the genetic signature of the sampled population in Portugal revealed that it is most likely an arrival from South America. The latest European countries to report *T. peregrinus* were Spain (2014)^57^, Albania (2016)^58^, and Greece (2017)^59^. The genetic diversity in these countries remains unknown, except for Spain, where haplotype A was reported in Madrid, with two main invasion routes being suggested: either an invasion from South America to Portugal and then an expansion to Spain; or that *T. peregrinus* invaded Spain from South America^22^.

For other European countries, we suggest that the bronze bug spread within the Mediterranean countries similarly to other invasive species that feed on eucalyptus trees. Possible invasion routes of *G. brimblecombei* into Europe were suggested from South or North America, considering the Spatio-temporal events and the presence of this insect in the New World since the 20^th^ century^60^. Subsequent events demonstrated the spread of *G. brimblecombei* to Mediterranean countries^61^. The connections between countries, mainly motorways and waterways linking Portugal, Spain, France, Italy, Greece, Algeria, and Morocco, were suggested as the main invasion routes of this pest^62^.

Route 6: The invasion of *T. peregrinus* to Reunion Island (2013)^23^ may have resulted from the flow of people and goods and the island's proximity to South Africa, where haplotype D is predominant^16^. However, a direct introduction from Australia is also possible based on distance and the presence of haplotype D in the site of origin of this species.

Route 7: The invasion of *T. peregrinus* into Israel possibly occurred from a country with the bordering to the Mediterranean Sea, where this species was already present, based on connections of motorways and waterways and the intense movement of people and goods between countries^60,62^. The recent expansion of eucalypts forestry in Asia could also influence the occurrence and detection of pests^1^, including the spread of *T. peregrinus* soon into new countries in Asia.

Routes 8 and 9: In North America, the first report of *T. peregrinus* was in Mexico in 2015^63^, ten years after its detection in Argentina. In 2016, this pest was found in the USA, in California in the greater Los Angeles area, specifically north Hollywood in the San Fernando Valley^64,65^, and Heartwell Park in Long Beach^65^. The first detection event might not be the actual first incursion and the establishment of a pest. Therefore, because of the short distance and the short period of this pest’s detection between Mexico and California (USA), it is impossible to know definitely in which country of North America *T. peregrinus* first entered. However, the presence of the same haplotype A in all areas of the Americas, suggests that a single invasion starting from South America and spreading northward to California might have occurred. Nevertheless, as before, Australia should also be considered as a source of *T. peregrinus* in North America because of the presence of haplotype A.

In general, in the USA, two invasion routes are considered dominant in the introduction of insects and diseases. Most harmful, non-native species (62%) likely entered North America with live plants and 30% probably arrived with wood packaging material (WPM) or other wood products^66^. In 2017, several specimens of *T. peregrinus* were found on *Eucalyptus* twigs in a shipment of cut flowers from Mexico that was intercepted at the port of Brownsville (Texas). Because *T. peregrinus* had quarantine status, the cut flowers returned to Mexico as a safety measure^67^. The twin ports of Los Angeles and Long Beach are the busiest in the USA and the international airports of San Francisco and Los Angeles are main entry points for passengers and air cargo^68^; all serve as gateways for pest introductions.

Our mtDNA analysis, including temporal and geographical sequence data, provides information that suggested scenarios of invasion routes of *T. peregrinus* in South America, North America, Europe, and Asia. These scenarios are an important source of information that assists in the planning and management of biosafety measures to prevent pests like *T. peregrinus*, from invading and colonizing new sites. Further studies using nuclear markers (e.g. microsatellites) or other mtDNA regions can aid to postulate new scenarios.

### Factors involved in the successful invasion and establishment of *T. peregrinus*

Several insect pests of eucalypts have undergone dispersal to two or more continents, for example, *L. invasa*, *Gonipterus* spp., and *Glycaspis brimblecombei* (Moore, 1964) (Hemiptera: Aphalaridae). Evidence exists that these invasive species possess and exploit mechanisms to aid in their dispersal^1^. In the specific case of *T. peregrinus*, adults do not show a strong natural ability to disperse except when aided by human activities^20^. However, nymphs are particularly mobile^8^ and could be more easily dispersed. Besides, biological factors account for the success of many invasive insects into a new location^69^, and we believe that certain biological characteristics of *T. peregrinus* represent an important portion of its invasion success throughout more than 20 countries. The life cycle of *T. peregrinus* is about 60 days and has a rather high potential for breeding (fecundity of 60 eggs/female) conditioning to reproduce more than twice a year and increase its population density^50,70^. Once this pest invades a new location, the lack of natural enemies facilitates its establishment and dispersion^71^ into the wide range of host trees of *Eucalyptus* and *Corymbia* (over 52 host species) found in many countries in the world^72^. *T. peregrinus* also thrives under a wide range of temperatures, varying from 4 °C to 34 °C, which explains its wide distribution and possible invasion into new areas in the future^73^. Its invasion success is also related to the small body size (± 3–4 mm)^9^ that facilitates its dispersal over long distances, whether actively or passively by the wind (draft)^74^ or by the movement of people or any kind of goods, especially timber, where is very difficult to be detected due to its small size.

Successful invaders are those featuring a small body size, several generations per year, long flight displacement and low incidence of diapause^69^. All these traits account for the rapid adaptation to new environmental conditions^75^. For example, the successful invasion of *L. invasa* into new areas was suggested to be a consequence of this pest's high dispersal capabilities^32^, strong resistance to low temperatures, the presence of eucalypt plantations worldwide^76^, and a lack of natural enemies in the invaded areas^77^. Moreover, synergic interactions among invasive species have been reported as a “meltdown” process that accelerates the impact of these species in the invaded area^78^. In line with this theory, both the eucalyptus lerp psyllid *G. brimblecombei* and *T. peregrinus* display a positive interaction that benefits from the preference of bronze bug females to oviposit on leaves bearing lerps^79^.

Considering the invasion success of haplotype A worldwide reported in this work and that the most common maternal lineages of *T. peregrinus* sampled in Australia, specifically in Sydney from 2001 to 2009, were haplotypes A, D, G and H^16,20^, the most likely source of *T. peregrinus* seems to be Sydney^16,20^ or Chelmsford^20^ for haplotypes A and D. The most commons haplotypes in a given population are more likely to be sampled and eventually to spread^16,43,80,81^, as it seems to have happened to haplotype A of *T. peregrinus*. On the other hand, haplotypes D, G and H were found restricted to some areas or not found outside its place of origin, although individuals might have dispersed to new areas but did not adapted and successfully establish as haplotype A.

The rate of invasion by insect pests in eucalypt plantations has increased approximately fivefold since the 1980s^1^. During our sampling of *T. peregrinus* specimens in over 28 locations nearly worldwide, we found that this insect was present in eucalypt trees along highways, and in parks, urban forests and landscapes, and commercial plantations. The growing flow of people among countries using diverse ways of transport and the unfamiliarity concerning the risks of transporting plant materials, either within or outside their natural areas, favor this invasive process.

## References

[1] Hurley BP (2016). Increasing numbers and intercontinental spread of invasive insects on eucalypts. Biol. Invasions..

[2] Paine TD, Steinbauer MJ, Lawson SA (2011). Native and exotic pests of Eucalyptus: a worldwide perspective. Annu. Rev. Entomol..

[3] Branco S, Videira N, Branco M, Paiva MR (2015). A review of invasive alien species impacts on eucalypt stands and citrus orchards ecosystem services: towards an integrated management approach. J. Environ. Manag..

[4] Carpintero DL, Dellapé PM (2006). A new species of *Thaumastocoris* Kirkaldy from Argentina (Heteroptera: Thaumastocoridae: Thaumastocorinae). Zootaxa.

[5] Button, G. Thaumastocoris peregrinus. NCT Forestry Co-operative Limited. http://www.nctforest.com/home.php?nav=pubs&pubtypeid=1&archive=1 (2007).

[6] Soliman EP (2012). Biology of *Thaumastocoris peregrinus* in different eucalyptus species and hybrids. Phytoparasitica.

[7] Wilcken, C. F. Percevejo bronzeado do eucalipto (*Thaumastocoris peregrinus*) (Hemiptera: Thaumastocoridae): ameaça às florestas de eucalipto brasileiras. IPEF Programa de proteção florestal - PROTEF/IPEF. http://www.ipef.br/protecao/alerta-percevejo.pdf (2008).

[8] Wilcken CF (2010). Bronze bug *Thaumastocoris peregrines* Carpintero and Dellapé (Hemiptera: Thaumastocoridae) on *Eucalyptus* in Brazil and its distribution. J. Plant Protect. Resear..

[9] Jacobs DH, Neser S (2005). *Thaumastocoris australicus* Kirkaldy (Heteroptera: Thaumastocoridae): a new insect arrival in South Africa, damaging to *Eucalyptus* trees: research in action. S. Afr. J. Sci..

[10] Noack AE, Coviella CE (2006). *Thaumastocoris australicus* Kirkaldy (Hemiptera: Thaumastocoridae): first record of this invasive pest of *Eucalyptus* in the Americas. Gen. Appl. Entomol..

[11] EPPO. Global database. Addition of *Thaumastocoris peregrinus* to the EPPO Alert List, https://gd.eppo.int/reporting/article-1974 (2012).

[12] Lesieur V (2019). The rapid spread of *Leptoglossus occidentalis* in Europe: a bridgehead invasion. J. Pest Sci..

[13] Seebens H (2017). No saturation in the accumulation of alien species worldwide. Nat. Communications.

[14] Westphal MI, Browne M, MacKinnon K, Noble I (2008). The link between international trade and the global distribution of invasive alien species. Biol. Invasions.

[15] Madden MJL (2019). Using DNA barcoding to improve invasive pest identification at U.S. ports-of-entry. Plos One.

[16] Nadel RL (2010). DNA bar-coding reveals source and patterns of *Thaumastocoris peregrinus* invasions in South Africa and South America. Biol. Invasions.

[17] Kang TH, Kim S, Hong KJ, Lee HS (2018). DNA barcoding in quarantine inspection: a case study on quarantine insect monitoring for Lepidoptera obtained through quarantine inspection on foreign vessels. Mitochondrial DNA Part B.

[18] Piper AM (2019). Prospects and challenges of implementing DNA metabarcoding for high-throughput insect surveillance. GigaScience.

[19] Staden RBKF, Bonfield JK (2000). The Staden package, 1998. Methods in Molecular Biology.

[20] Lo N (2019). Population genetics of the Australian eucalypt pest *Thaumastocoris peregrinus*: evidence for a recent invasion of Sydney. J. Pest Science.

[21] Nugnes, F., Caprio, E., di Prisco, G., Laudonia, S. & Sasso, R. *Thaumastocoris peregrinus* Carpintero & Dellapé (Heteroptera, Thaumastocoridae): a new haplotype in Italy. In: XXIV Italian National Congress of Entomology, Orosei, 2014, p. 98. 10.13140/2.1.3217.5045 (2014).

[22] Machado Dayanna Do Nascimento, Costa Ervandil Corrêa, Perini Clérison Régis, Ugalde Gustavo Andrade, Saldanha Mateus Alves, Leitão João Vitor, Colpo Tiago Lovato, Arnemann Jonas André, Rivera Adolfo Cordero (2019). The ongoing dispersion of the Eucalyptus bronze bug (Thaumastocoris peregrinus) in Spain. Forest Systems.

[23] Streito JC (2016). Report of the invasive species *Thaumastocoris peregrinus* Carpintero & Dellapé, 2006, on Réunion Island (Hemiptera, Heteroptera, Thaumastocoridae). Bul. Soc. Entomol. France.

[24] Clement M, Posada DC, Crandall KA (2000). TCS: a computer program to estimate gene genealogies. Molecular Ecology.

[25] Leigh JW, Bryant D (2015). Popart: full-feature software for haplotype network construction. Methods in Ecology and Evolution.

[26] Puillandre N (2008). Genetic bottleneck in invasive species: the potato tuber moth adds to the list. Biol. Invasions.

[27] Caron V, Norgate M, Ede FJ, Nyman T, Sunnucks P (2013). Novel microsatellite DNA markets indicate strict parthenogenesis and few genotypes in the invasive willow saw fly *Nematus oligospilus*. Bull. Entomol. Res..

[28] Rollins LA (2009). Invasive species can’t cover their tracks: using microsatellites to assist management of starling (*Sturnus vulgaris*) populations in Western Australia. Mol. Ecol..

[29] Nei M, Maruyama T, Chakraborty R (1975). The bottleneck effect and genetic variability in populations. Evolution.

[30] Carter M, Smith M, Harrison R (2010). Genetic analyses of the Asian longhorn beetle (Coleoptera, Cerambycidae, *Anoplophora glabripennis*), in North. Biol. Invasions.

[31] Nugnes F (2015). Genetic Diversity of the Invasive Gall Wasp *Leptocybe Invasa* (Hymenoptera: Eulophidae) and of its *Rickettsia* endosymbiont, and associated sex-ratio differences. PloS One.

[32] Dittrich-Schröder G (2018). Population genetic analyses of complex global insect invasions in managed landscapes: a *Leptocybe invasa* (Hymenoptera) case study. Biol. Invasion.

[33] Ascunce MS (2011). Global invasion history of the fire ant *Solenopsis invicta*. Science.

[34] Gotzek D (2015). Global invasion history of the tropical fire ant: a stowaway on the first global trade routes. Mol. Ecol..

[35] Boissin E (2012). Retracing the routes of introduction of invasive species: the case of the *Sirex noctilio* woodwasp. Mol. Ecol..

[36] Dlugosch KM, Parker IM (2008). Founding events in species invasions: genetic variation, adaptive evolution, and the role of multiple introductions. Mol. Ecology.

[37] Garnas JR (2016). Complex patterns of global spread in invasive insects: eco-evolutionary and management consequences. Biol. Invasions.

[38] Essl F (2015). Crossing frontiers in tackling pathways of biological invasions. Biosci..

[39] Lopes da Silva M (2014). The Role of Natural and Human-Mediated Pathways for Invasive Agricultural Pests: A Historical Analysis of Cases from Brazil. Agricul. Sci..

[40] Goldstien Sharyn J., Dupont Lise, Viard Frédérique, Hallas Paul J., Nishikawa Teruaki, Schiel David R., Gemmell Neil J., Bishop John D. D. (2011). Global Phylogeography of the Widely Introduced North West Pacific Ascidian Styela clava. PLoS ONE.

[41] Townsend, C. R., Begon, M. & Harper, J. L. Fundamentos em Ecologia. 3 Ed. Artmed, Porto Alegre (2010).

[42] Vincek V (1997). How large was the founding population of Darwin's finches?. Proc. Biol. Sci..

[43] Ficetola GF, Bonin A, Miaud C (2008). Population genetics reveals origin and number of founders in a biological invasion. Mol. Ecol..

[44] Dickey AM (2013). Population Genetics of Invasive *Bemisia tabaci* (Hemiptera: Aleyrodidae) Cryptic Species in the United States Based on Microsatellite Markers. J. Econ. Entomol..

[45] Tay TW (2017). Mitochondrial DNA and trade data support multiple origins of *Helicoverpa armigera* (Lepidoptera, Noctuidae) in Brazil. Scientific Reports.

[46] Yunke W (2017). Identification of wood-boring beetles (Cerambycidae and Buprestidae) intercepted in tradeassociated solid wood packaging material using DNA barcoding and morphology. Scientific Reports.

[47] Arnemann JA (2016). Complete mitochondrial genome of the soybean stem fly *Melanagromyza sojae* (Diptera: Agromyzidae). Mitochondrial DNA Part A.

[48] Arnemann, J. A. *et al*. Multiple incursion pathways for *Helicoverpa armigera* in Brazil show its genetic diversity spreading in a connected world. *Scientific Reports* **9**, 10.1101/762229 (2019). 10.1038/s41598-019-55919-9 PMC6920452 31852963

[49] Mayorga SI, Ruiz CG, Sandoval AC, Valenzuela JE (2011). Detection of *Thaumastocoris peregrinus* (Hemiptera: Thaumastocoridae) associated to *Eucalyptus* spp. in Chile. (Detección de *Thaumastocoris peregrinus* (Hemiptera: Thaumastocoridae) asociado a *Eucalyptus* spp. en Chile. Bosque.

[50] Noack AE, Rose HA (2007). Life-history of *Thaumastocoris peregrinus* and *Thaumastocoris* sp. In the laboratory with some observations on behavior. Gen. App. Entomol..

[51] Roy BA (2014). Increasing forest loss worldwide from invasive pests requires new trade regulations. Front. Ecol. Environ..

[52] Withers TM (2001). Colonization of eucalypts in New Zealand by Australian insects. Aust. Ecol..

[53] Laudonia S, Sasso R (2012). The bronze bug *Thaumastocoris peregrinus*: a new insect recorded in Italy, damaging to *Eucalyptus* trees. Bul. Insectology.

[54] Garcia A, Figueiredo E, Valente C, Monserrat VJ, Branco M (2013). First record of *Thaumastocoris peregrinus* in Portugal and of the Neotropical predator *Hemerobius bolivari* in. Europe. Bul. Insectology.

[55] UNECE, FAO. Forest products: annual market review 2004-2005, Timber Bulletin, volume 58 (2005). United Nations Economic Comission for Europe, Food and Agriculture Organization, Geneva, Switzerland. http://www.ipef.br/estatisticas/relatorios/FAO-forest_products-2005.pdf (2005).

[56] UNECE, FAO. Forest products: annual market review 2011-2012. United Nations Economic Comission for Europe, Food and Agriculture Organization, Geneva, Switzerland. http://www.unece.org/fileadmin/DAM/timber/publications/FPAMR_2012.pdf (2012).

[57] Vivas L, Crespo J, Jacinto V (2015). Primer registro de la especie invasora *Thaumastocoris peregrinus* Carpintero & Dellapé, 2006 en España y nuevos datos para Portugal (Hemiptera: Thaumastocoridae). BV News.

[58] Heyden TVD (2017). The first record of *Thaumastocoris peregrinus* Carpintero & Dellapé, 2006 (Hemiptera: Heteroptera: Thaumastocoridae) for Albania. Rev. Gad. Entomol..

[59] Petrakis PV (2018). First record of the bug *Thaumastocoris peregrinus* in Greece. Entomologia Hellenica.

[60] Brennan EB, Gill RJ, Hrusa GF, Weinbaum SA (1999). First record of *Glycaspis brimblecombei* (Moore) (Homoptera: Psyllidae) in North America: initial observations and predator associations of a potentially serious new pest of Eucalyptus in California. *Pan-Pacific*. Entomol.

[61] Dhahri S, Ben Jamaa ML, Garcia A, Boavida C, Branco M (2014). Presence of *Glycaspis brimblecombei* and its Parasitoid *Psyllaephagus bliteus* in Tunisia and Portugal. Silva Lusit..

[62] Reguia K, Peris-Felipo FJ (2013). *Glycaspis brimblecombei* Moore,1964 (Hemiptera Psyllidae) invasion and new records in the Mediterranean area. Biodiversity J..

[63] Jiménez-Quiroz E, Vanegas-Rico JM, Morales-Martínez O, Lomeli-Flores JR, Rodríguez-Leyva E (2016). First Record of the Bronze Bug, *Thaumastocoris peregrinus* Carpintero & Dellapé 2006 (Hemiptera: Thaumastocoridae), in Mexico. J. Agric. Urban Entomol..

[64] BugGuide. 2016. *Thaumastocoris peregrinus*. On-line, http://bugguide.net/node/view/1236781 (2019).

[65] Hodel DR, Arakelian G, Ohara LM (2016). The Bronze Bug Another New Threat to Eucalypts in California. PalmArbor.

[66] Liebhold AM, Brockerhoff EG, Garrett LJ, Parke JL, Britton KO (2012). Live plant imports: the major pathway for forest insect and pathogen invasions of the US. Front. Ecol. Environ..

[67] U. S. Department of Homeland Security. Brownsville Port of Entry CBP Agriculture Specialist Intercepts First in Port Pest in Eucalyptus Branches. https://www.cbp.gov/newsroom/local-media-release/brownsville-port-entry-cbp-agriculture-specialist-intercepts-first-1 (2017).

[68] Paine TD, Millar JG, Daane KM (2010). Accumulation of Pest Insects on Eucalyptus in California: random Process or Smoking Gun. J. Econ. Entomol..

[69] EPPO. Global Database. Distribution *Thaumastocoris peregrinus*, https://gd.eppo.int/taxon/THMCPE/distribution (2018).

[70] CABI. Invasive Species Compendium. *Thaumastocoris peregrinus* (bronze bug). https://www.cabi.org/isc/datasheet/109741#20123208401 (2018).

[71] Chilima, C. Z. New *Eucalyptus* Pest Recorded from Zimbabwe. Forest Invasive Species Network for Africa (FAO) [WWW document]. http://www.fao.org/forestry/fisna/26061/en/ (2007).

[72] Nahrung HF, Swain AJ (2015). Strangers in a strange land: do life history traits differ for alien and native colonisers of novel environments?. Biol. Invasions.

[73] Nadel Ryan L., Wingfield Michael J., Scholes Mary C., Garnas Jeffrey R., Lawson Simon A., Slippers Bernard (2014). Population dynamics of Thaumastocoris peregrinus in Eucalyptus plantations of South Africa. Journal of Pest Science.

[74] Corallo Belén, Simeto Sofia, Martínez Gonzalo, Gómez Demian, Abreo Eduardo, Altier Nora, Lupo Sandra (2019). Entomopathogenic fungi naturally infecting the eucalypt bronze bug, Thaumastocoris peregrinus (Heteroptera: Thaumastocoridae), in Uruguay. Journal of Applied Entomology.

[75] Saavedra MC, Withers TM, Holwell GI (2015). Susceptibility of four *Eucalyptus* host species for the development of *Thaumastocoris peregrinus* Carpintero and Dellapé (Hemiptera: Thaumastocoridae). Forest Ecol. Manag..

[76] Saavedra MC, Avila GA, Withers TM, Holwell GI (2015). The potential global distribution of the Bronze bug *Thaumastocoris peregrinus* Carpintero and Dellapé (Hemiptera: Thaumastocoridae). Agr. Forest Entomol..

[77] Osborne, J. L.; Loxdale, H. D. & Woiwod, I. P. Monitoring insect dispersal: methods and approaches. In: Bullock, J. M., Kenward, R. E and Hails, R. S. (Eds.). Dispersal Ecology: 42nd Symposium of the British Ecological Society, P. 24–49 (Cambridge University Press, 2002).

[78] Whitney KD, Gabler CA (2008). Rapid evolution in introduced species, ‘invasive traits’ and recipient communities: challenges for predicting invasive potential. Diversity distrib..

[79] Zheng XL (2014). A review of invasive biology, prevalence and management of *Leptocybe invasa* Fisher & La Salle (Hymenoptera: Eulophidae: Tetrastichinae). Afr. Entomol..

[80] Protasov A, Doganlar M, Salle JL, Mendel Z (2008). Occurrence of two local *Megastigmus* species parasitic on the eucalyptus gallwasp *Leptocybe invasa* in Israel and Turkey. Phytoparasitica.

[81] Simberloff D, Holle BV (1999). Positive interactions of nonindigenous species: invasional meltdown?. Biol. Invasions.

[82] Martínez G, González A, Dicke M (2018). Effect of the eucalypt lerp psyllid *Glycaspis brimblecombei* on adult feeding, oviposition‐site selection, and offspring performance of the bronze bug, *Thaumastocoris peregrinus*. Entomol. Experim. Appl..

[83] Sakai AK (2001). The population biology of invasive species. Ann. Rev. Ecol. Syst..

[84] Roderick GK (1996). Geographic structure of insect populations: gene flow, phylogeography, and their uses. Ann. Rev. Entomol..

[85] Martínez G, Bianchi M (2010). Primer registro para Uruguay de la chinche del eucalipto, *Thaumastocoris peregrinus* Carpintero y Dellappé, 2006 (Heteroptera: Thaumastocoridae). Agrociencia.

[86] Díaz EAB, Coronel RS, Godziewski D (2013). Consideraciones sobre dos nuevas plagas del eucalipto em Paraguay. El psílido de la concha o escudo Glycaspis brimblecombei (Hemiptera: psyllidae) y La chinche marrón Thaumastocoris peregrinus (Hemiptera: Thaumastocoridae). Bol. Museo Nac. Hist. Nat. Parag..

[87] Sopow S, George S, Ward N (2012). Bronze bug, *Thaumastocoris peregrinus*: a new *Eucalyptus* pest in New Zealand. Surveillance.

[88] Carapezza A (2014). The arrival of one more eucalyptus pest in Sicily: *Thaumastocoris peregrinus* Carpintero & Dellapé, 2006 (Hemiptera Heteroptera Thaumastocoridae). Naturalista Sicil..

[89] Suma P, Nucifora S, Bella S (2014). New distribution record of the invasive bronze bug *Thaumastocoris peregrinus* Carpintero and Dellapé (Heteroptera, Thaumastocoridae) in Italy. Bull. OEPP/EPPO.

[90] Novoselsky T, Freideberg A (2016). First record of *Thaumastocoris peregrinus* (Hemiptera: Thaumastocoridae) in the Middle East, with biological notes on its relations with eucalyptus trees. Isr. J. Entomol..

